# Optimal Control of Time-Delay Fractional Equations via a Joint Application of Radial Basis Functions and Collocation Method

**DOI:** 10.3390/e22111213

**Published:** 2020-10-26

**Authors:** Shu-Bo Chen, Samaneh Soradi-Zeid, Hadi Jahanshahi, Raúl Alcaraz, José Francisco Gómez-Aguilar, Stelios Bekiros, Yu-Ming Chu

**Affiliations:** 1 School of Science, Hunan City University, Yiyang 413000, China; shubo.chen@163.com; 2 Faculty of Industry and Mining (khash), University of Sistan and Baluchestan, Zahedan 98155-987, Iran; soradizeid@eng.usb.ac.ir; 3 Department of Mechanical Engineering, University of Manitoba, Winnipeg, MB R3T 5V6, Canada; jahanshahi.hadi90@gmail.com; 4 Research Group in Electronic, Biomedical and Telecommunication Engineering, University of Castilla-La Mancha (UCLM), 16071 Cuenca, Spain; 5 CONACyT-Tecnológico Nacional de México/CENIDET, Interior Internado Palmira S/N, Col. Palmira, Cuernavaca C.P. 62490, Morelos, Mexico; jose.ga@cenidet.tecnm.mx; 6 Department of Economics, European University Institute, Via delle Fontanelle, 18, I-50014 Florence, Italy; stelios.bekiros@eui.eu; 7 Rimini Centre for Economic Analysis (RCEA), LH3079, Wilfrid Laurier University, 75 University Ave W., Waterloo, ON N2L3C5, Canada; 8 Department of Mathematics, Huzhou University, Huzhou 313000, China; 9 Hunan Provincial Key Laboratory of Mathematical Modeling and Analysis in Engineering, Changsha University of Science & Technology, Changsha 410114, China

**Keywords:** fractional optimal control problem, delay system, radial basis function, direct optimization, collocation points, nonlinear programming problem

## Abstract

A novel approach to solve optimal control problems dealing simultaneously with fractional differential equations and time delay is proposed in this work. More precisely, a set of global radial basis functions are firstly used to approximate the states and control variables in the problem. Then, a collocation method is applied to convert the time-delay fractional optimal control problem to a nonlinear programming one. By solving the resulting challenge, the unknown coefficients of the original one will be finally obtained. In this way, the proposed strategy introduces a very tunable framework for direct trajectory optimization, according to the discretization procedure and the range of arbitrary nodes. The algorithm’s performance has been analyzed for several non-trivial examples, and the obtained results have shown that this scheme is more accurate, robust, and efficient than most previous methods.

## 1. Introduction

In the last years, the use of fractional calculus has increased significantly due to its attractive applications in physical and engineering systems [1,2,3], materials [4], biology [5], finance [6], and so on. Moreover, fractional differential equations (FDEs) have also recently gained considerable importance in pure and applied mathematics [7], engineering [8], physics [9], and bio-systems [10]. Nonetheless, despite this growing variety of applications, it is often difficult to find numerical methods with low computing cost and enough accuracy for resolving these kinds of equations and analytically handling solutions in many problems. Thus, numerous methods to deal with that purpose have been proposed in the last decade, including spectral tau method [11], fractional power series [12], and fractional-order Legendre wavelet Tau method [13].

Combining an optimal control problem and fractional calculus, which is well-known as a fractional optimal control problem (FOCP), is one of the latest exciting challenges among mathematical researchers. Indeed, a FOCP is an optimal control problem where the dynamical system is governed by FDEs. To address this challenge, recent studies suggest the use of radial basis functions [14] and the spectral tau algorithm [15]. More details about practical approximation techniques for solving FDEs and FOCPs can be found in the fresh review articles in [16,17], respectively.

After physical realization of FOCPs in very diverse scenarios, many researchers have lately been fascinated by time delay fractional dynamics in fields such as electronic, biological, and transport systems. Because FDEs with time delay are difficult form of differential equations, potent, and novel numerical methods for their resolution are necessary. Despite its complexity, the analysis of delay differential equations is one of the most exciting topics that have been taken widespread attention among researchers and have been incorporated in models with infinite dimensions in multiple areas. However, there are still few works devoted to obtain numerical solutions for delay differential equations of fractional order. Among these works, we can mention those proposing the use of radial basis functions [18], Müntz–Legendre wavelet transform [19], Picard iteration [20], and piecewise fractional-order Taylor functions [21]. A time delay FOCP (DFOCP) is defined when the dynamic system is governed by previous information at the specified time. In other words, time delay systems result when traditional point-wise modeling assumptions are replaced by realistic distributed ones. The basic fact reflected by the specific mathematical model with time delay is that the change of trajectory about time *t* not only depends on the *t* moment itself, but it is also affected by some certain conditions before, even the reflection of some certain factors before, that moment. This kind of circumstance is abundant in the objective world. For example, knowing previous information about predators and even prey, instead of considering the current level of the predator model, can directly affect on the birth rate. The fractional derivatives capture the history of the variable, i.e., have memory, contrary to integer-order derivatives, which are local operators. This characteristic makes them an important tool in the modeling of memory-intense and delay systems. Therefore, DFOCPs are used to model phenomena which have memory, as well as realistic distribution hypotheses. One of the well-known models that can be applied in the classical and quantum mechanics is the harmonic oscillator, which is described as an DFOCP [22,23,24].

Motivated by the numerous recent applications of DFOCPs, the solution of these kinds of problems has been of considerable concern for researchers. Over the last decade, many scholars have worked on the numerical investigation of DFOCPs, proposing algorithms such as Bernstein polynomials [25], shifted Legendre orthonormal polynomials [26], Chelyshkov wavelets [27], Bernoulli wavelets [28], Boubaker functions [29], measure theory approach [30,31], and Legendre wavelets [32]. Unfortunately, these methods present a high computational cost in discretization of the fractional terms. Thus, the use of global schemes, such as radial basis functions (RBFs) approaches, seems to be a more appropriate alternative, as they are more helpful tools in discretizing fractional calculus. However, direct methods are widely applied for solving fractional problems by first using approximation and afterwards discretization to the original problem. Moreover, by means of some parameterization of the state and/or control variables, direct optimization methods can transcribe an infinite-dimensional continuous problem to a finite-dimensional ones. Within this context, a new direct computational method is introduced in the present work, which uses RBFs for solving DFOCPs. Our proposed approach employs any global RBFs (e.g., Gaussian RBFs, multiquadrics, inverse multiquadrics, etc.) to approximate the state and control variables fo the problem. As well, arbitrary discretization nodes (e.g., equally-spaced nodes, orthogonal nodes, etc.) are used to convert the DFOCP into a nonlinear programming problem (NLP) with unknown coefficients. This approach with any global RBFs for parameterization and any arbitrary points for discretization, has been able to provide a very applicable framework for solving DFOCPs. The practical importance of the proposed method is that a variety of RBF functions can be applied for interpolation of states and controls, instead of being limited to a specific type of polynomial as in polynomial-based methods. Moreover, a wide range of arbitrary nodes can also be easily employed for discretization of the fractional terms, thus resulting in a flexible RBF framework for solving DFOCPs.

The outline of this paper is as follows. Section 2 demonstrates the problem statement and the basic concepts about fractional derivative. Some preliminaries of RBFs for subsequent developments are presented in Section 3. Moreover, we present a direct RBF collocation scheme to solve DFOCPs in this section. The numerical results obtained by the proposed approach for some non-trivial examples are described and compared with other previous works in Section 4. Finally, the most relevant conclusions are summarized in Section 5, along with some future perspectives.

## 2. Statement of the Problem

The aforementioned performance of meshless methods have encouraged some researchers to develop new computing architectures and techniques where the primary focus was on hardware simplicity. In order to lower the implementation cost, we want to explore an applicable numerical scheme to find the approximate solutions of the following DFOCP,

(1) $$J=\frac{1}{2}\int_{t_{0}}^{t_{f}}\left(r\left(t\right)x^{2}\left(t\right)+q\left(t\right)u^{2}\left(t\right)\right)dt,$$

subject to dynamic constraints,

(2) $$\begin{array}{c} D^{\alpha }x\left(t\right)=a\left(t\right)x\left(t\right)+e\left(t\right)u\left(t\right)+b\left(t\right)x\left(t-\eta \right)+f\left(t\right)u\left(t-\delta \right)+g\left(t\right),\\ x\left(t\right)=\varphi_{1}\left(t\right),\;t_{0}-\eta \leq t\leq t_{0},\\ u\left(t\right)=\varphi_{2}\left(t\right),\;t_{0}-\delta \leq t\leq t_{0}, \end{array}$$

where $x\left(t\right)\in C^{1-\alpha }\left[t_{0},t_{f}\right]$ is the state variable in which

$$C^{n-\alpha }\left[t_{0},t_{f}\right]=\{x:\left[t_{0},t_{f}\right]\to R^{n}\;:\frac{d^{n-1}}{dt^{n-1}}\left(D^{\alpha }x\left(t\right)\in L_{1}\left[t_{0},t_{f}\right],\;\;n-1<\alpha <n\right\},$$

and u(t)∈U mentions the control variable, in which $U\subset R^{m}$ represents the set of continuous functions. Furthermore, it is assumed that $J\in C^{1}\left[t_{0},t_{f}\right]$, η>0, $\delta <t_{f}-t_{0}$, 0<α≤1. In addition, a(t), b(t), e(t), f(t), and g(t) are continuous functions; $\varphi_{1}\left(t\right)$ and $\varphi_{2}\left(t\right)$ are known functions; and r(t) and q(t) are two symmetric positive semidefinite and definite matrixes, respectively, which show the time-varying coefficients of the state and control variables in the cost function with continuous functions. Moreover, it is assumed that the dynamic system (2) is at rest from −∞ to $t_{0}-\eta$. Furthermore, $D^{\alpha }$ is the fractional differentiation operator of order α that is defined as follows.

**Definition** **1.** *For a given function f(t) and α>0, n−1<α≤n, n∈N, the operators*

(3) $${}_{t_{0}}D_{t}^{\alpha }f\left(t\right)=\frac{1}{\Gamma (n-\alpha )}\int_{t_{0}}^{t}{\left(t-\tau \right)}^{n-\alpha -1}f^{\left(n\right)}\left(\tau \right)d\tau ,$$

*and*

(4) $${}_{t}D_{t_{f}}^{\alpha }f\left(t\right)=\frac{{\left(-1\right)}^{n}}{\Gamma (n-\alpha )}\int_{t}^{t_{f}}{\left(\tau -t\right)}^{n-\alpha -1}f^{\left(n\right)}\left(\tau \right)d\tau ,$$

*are called, respectively, the left and right Caputo fractional derivatives (CFDs) of order α>0.*

Furthermore,

(5) $$\begin{array}{c} D^{\alpha }K=0,\;\left(K\;is\;a\;constant\right),\\ D^{\alpha }t^{\beta }=\frac{\Gamma (\beta +1)}{\Gamma (\beta -\alpha +1)}x^{\beta -\alpha },\;\beta >\alpha -1,\\ D^{\alpha }\left(\lambda f\left(t\right)+\mu g\left(t\right)\right)=\lambda D^{\alpha }f\left(t\right)+\mu D^{\alpha }g\left(t\right). \end{array}$$

The aforementioned properties of CFDs have led us to use this definition in the following. The main contribution of this paper is thus to suggest a direct method based on RBFs and collocation points to obtain the optimal values of u(t) and x(t), $t\in [t_{0},t_{f}]$, satisfying Equation (2) and minimizing the quadratic performance index in Equation (1). One advantage of this method is that it does not use the maximum principle and calculate pontryagin variations to solve the problem, so there is no need for analytical separation of cost and constraint statements. Moreover, in general terms, the direct methods (such as the proposed one) have a greater convergence radius than indirect methods [33,34]. Moreover, to make the problem significantly simpler, we have tried to reformulated the DFOCP expressed in Equations (1) and (2) as an equivalent NLP by making use of the interpolate approximate of basis functions.

## 3. Method of Solution

In this section, a brief description of the proposed method to directly solve the DFOCP modeled by Equations (1) and (2) is introduced.

### 3.1. RBF Definition and Collocation Method

Any function Φ that satisfies Φ(x)=ϕ(|x|), with ϕ∈C[0,∞), is a radial function. This function is positive definite or *m*-order conditionally positive definite on $R^{n}$, when

$$\sum_{i=1}^{N}\sum_{j=1}^{N}a_{i}a_{j}\Phi \left(x_{j}-x_{i}\right)>0,$$

in which all nonzero $a\in R^{n}$ satisfying $\sum_{i=1}^{N}a_{i}p\left(x_{i}\right)=0$, for all $p\in \Pi_{m}$, and $\Pi_{m}$ is the set of polynomials of degree m−1 or less. The primal space related to the nodal points $X_{N}$ is constructed as follows,

$$W_{N}=\left\{\sum_{i=1}^{N}a_{i}\Phi_{i}\left(x\right)\;\mathrm{such}\;\mathrm{that}\;\sum_{i=1}^{N}a_{i}p\left(x\right)=0,\;\forall p\in \Pi_{m-1}\right\}+\Pi_{m-1}.$$

Furthermore, each $u\in W_{N}$ can be shown as $u\left(x\right)=\sum_{i=1}^{N}a_{i}\Phi_{i}\left(x\right)+\sum_{j=1}^{N(m)}b_{j}p_{j}\left(x\right)$, where $p_{j}\left(x\right)$’s are monomial polynomials in $\Pi_{m-1}$.

Commonly used types of RBFs include the following forms, in which $r=∥x-x_{i}∥$ and the shape parameter ε controls their flatness [35].

- Piecewise Smooth:
  • $\phi \left(r\right)=r^{3}$,         Cubic RBF;
  • $\phi \left(r\right)=r^{5}$,         Quintic RBF;
  • $\phi \left(r\right)=r^{2}log\left(r\right)$,     Thin Plate spline (TPS) RBF;
  • $\phi \left(r\right)={\left(1-r\right)}^{m}+p\left(r\right)$,  Wendland functions where *p* is a polynomial.
- Infinitely Smooth:
  • $\phi \left(r\right)=\sqrt{1+{\left(\varepsilon r\right)}^{2}}$,     Multiquadric (MQ) RBF;
  • $\phi \left(r\right)=\frac{1}{1+{\left(\varepsilon r\right)}^{2}}$,     Inverse Quadratic (IQ) RBF.
  • $\phi \left(r\right)=e^{-{\left(\varepsilon r\right)}^{2}}$,       Gaussian RBF.

Now, we briefly introduce the RBFs collocation method. Let $\Omega \subseteq R^{d}$ and consider a boundary value problem as follows,

(6) Lu=finΩ,

(7) u=gon∂Ω,

where *L* is a linear differential operator and *d* is the dimension of the problem. We distinguish in our notation center $X=\{x_{1},...,x_{N}\}$ and the collocation points $\Xi =\{\alpha_{1},...,\alpha_{N}\}$. Then, we have the approximate solution of Equations (6) and (7) in the form

(8) $$\tilde{u}\left(x\right)=\sum_{i=1}^{N}\lambda_{i}\phi (∥x-x_{i}∥),$$

where $\lambda_{i}$, i=1,2,⋯,N, are unknown coefficients that determined by collocation, ϕ is a RBF, ∥.∥ is the Euclidean norm and $x_{i}$ is the centers of the RBFs.

Now, let Ξ divided into two subsets. One subset contains $N_{I}$ centers, $\Xi_{1}$, where Equation (6) is enforced and the other subset contains $N_{B}$ centers, $\Xi_{2}$, where boundary conditions are enforced. The collocation matrix is obtained by applying the collocation points to differential equation, and its boundary condition is as follows,

$$A=\left[\begin{array}{c} A_{I}\\ A_{B} \end{array}\right],$$

in which $A_{I}=L\phi (∥\alpha -x_{j}{∥)}_{\alpha =\alpha_{i}}$, $\alpha_{i}\in \Xi_{1}$, $x_{j}\in X$, and $A_{B}=L\phi (∥\alpha -x_{j}{∥)}_{\alpha =\alpha_{i}}$, $\alpha_{i}\in \Xi_{2}$, $x_{j}\in X$. By solving the linear system Aλ=F, we can obtain the unknown coefficients $\lambda_{i}$, in which *F* is a vector included $f(\alpha_{i})$, $\alpha_{i}\in \Xi_{1}$, and $g(\alpha_{i})$, $\alpha_{i}\in \Xi_{2}$.

### 3.2. Application of RBF Collocation Method

For solving a DFOCP by the RBF collocation method, without loss of generality, it has to be assumed that η≤δ. Then, we can rewrite the problem expressed in Equations (1) and (2) as follows,

(9) $$J=\frac{1}{2}\int_{t_{0}}^{t_{f}}\left(r\left(t\right)x^{2}\left(t\right)+q\left(t\right)u^{2}\left(t\right)\right)dt,$$

subject to

(10) $$\begin{array}{c} D^{\alpha }x\left(t\right)-a\left(t\right)x\left(t\right)-e\left(t\right)u\left(t\right)=b\left(t\right)\varphi_{1}\left(t-\eta \right)+f\left(t\right)\varphi_{2}\left(t-\delta \right)+g\left(t\right),\;t_{0}<t\leq \eta \\ D^{\alpha }x\left(t\right)-a\left(t\right)x\left(t\right)-e\left(t\right)u\left(t\right)-b\left(t\right)x\left(t-\eta \right)=f\left(t\right)\varphi_{2}\left(t-\delta \right)+g\left(t\right),\;\;\eta <t\leq \delta \\ D^{\alpha }x\left(t\right)-a\left(t\right)x\left(t\right)-e\left(t\right)u\left(t\right)-b\left(t\right)x\left(t-\eta \right)-f\left(t\right)u\left(t-\delta \right)=g\left(t\right),\;\;\delta <t\leq t_{f}\\ x\left(t_{0}\right)=\varphi_{1}\left(t_{0}\right),\\ u\left(t_{0}\right)=\varphi_{2}\left(t_{0}\right). \end{array}$$

For simplicity and clarity, the method is only derived for Cubic RBFs and equally spaced nodes. Therefore, we choose the same number of RBF functions and collocation points (*N*) for the following approximation,

(11) $$x\left(t\right)\approx \sum_{j=1}^{N}\lambda_{j}\phi (∥t-t_{j}∥),\;\mathrm{and}$$

(12) $$u\left(t\right)\approx \sum_{j=1}^{N}\gamma_{j}\phi (∥t-t_{j}∥).$$

Also, for the delay terms we have:

(13) $$x\left(t-\eta \right)\approx \sum_{j=1}^{N}\lambda_{j}\phi (∥t-\eta -t_{j}∥),\;\mathrm{and}$$

(14) $$u\left(t-\delta \right)\approx \sum_{j=1}^{N}\gamma_{j}\phi (∥t-\delta -t_{j}∥).$$

Now, fractional derivation from the sides of Equation (11) with respect to *t* yields

(15) $$D^{\alpha }x\left(t\right)\approx \sum_{j=1}^{N}\lambda_{j}D^{\alpha }\phi (∥t-t_{j}∥).$$

Obtaining a closed form analytic expression for the fractional derivative of a radial function may lead to a challenge. Accordingly, Mohammadi and Schaback [36] provided the required formulas for the fractional derivatives of RBFs, which allow us to use high order approximation methods for solving fractional problems. Now, we can approximate the continuous cost function described in Equation (9) with a trapezoidal quadrature rule as follows,

(16) $$J=\frac{1}{2}\sum_{i=1}^{N}w_{i}\left(r\left(t_{i}\right)x^{2}\left(t_{i}\right)+q\left(t_{i}\right)u^{2}\left(t_{i}\right)\right),$$

where $w_{i}$ and $t_{i}$ are weight and nodes of integral quadrature rule, respectively. Now, by substituting Equations (11)–(15) into the problem modeled in Equations (9) and (10) and evaluating the dynamic constraints expressed in Equation (10) at the collocation nodes, we have the following NLP problem,

(17) $$J=\frac{1}{2}\sum_{i=1}^{N}w_{i}\left[r\left(t_{i}\right){\left(\sum_{j=1}^{N}\lambda_{j}\phi (∥t_{i}-t_{j}∥)\right)}^{2}+q\left(t_{i}\right){\left(\sum_{j=1}^{N}\gamma_{j}\phi (∥t_{i}-t_{j}∥)\right)}^{2}\right],$$

subject to

(18) $$\begin{array}{cc} \sum_{j=1}^{N} & \lambda_{j}\left(D^{\alpha }\phi (∥t_{i}-t_{j}∥)-a\left(t_{i}\right)\phi (∥t_{i}-t_{j}∥)\right)-\sum_{j=1}^{N}\gamma_{j}\left(e\left(t_{i}\right)\phi (∥t_{i}-t_{j}∥)\right)\\ & =b\left(t_{i}\right)\varphi_{1}\left(t_{i}-\eta \right)+f\left(t_{i}\right)\varphi_{2}\left(t_{i}-\delta \right)+g\left(t_{i}\right),\;t_{0}<t_{i}\leq \eta \end{array}$$

$$\begin{array}{cc} \sum_{j=1}^{N} & \lambda_{j}\left(D^{\alpha }\phi (∥t_{i}-t_{j}∥)-a\left(t_{i}\right)\phi (∥t_{i}-t_{j}∥)-b\left(t_{i}\right)\phi (∥t_{i}-\eta -t_{j}∥)\right)\\ & -\sum_{j=1}^{N}\gamma_{j}\left(e\left(t_{i}\right)\phi (∥t_{i}-t_{j}∥)\right)=f\left(t_{i}\right)\varphi_{2}\left(t_{i}-\delta \right)+g\left(t_{i}\right),\;\eta <t_{i}\leq \delta \end{array}$$

$$\begin{array}{cc} \;\;\;\sum_{j=1}^{N} & \lambda_{j}\left(D^{\alpha }\phi (∥t_{i}-t_{j}∥)-a\left(t_{i}\right)\phi (∥t_{i}-t_{j}∥)-b\left(t_{i}\right)\phi (∥t_{i}-\eta -t_{j}∥)\right)\\ & -\sum_{j=1}^{N}\gamma_{j}\left(e\left(t_{i}\right)\phi (∥t_{i}-t_{j}∥)-f\left(t_{i}\right)\phi (∥t_{i}-\delta -t_{j}∥)\right)=g\left(t_{i}\right),\;\delta <t_{i}\leq t_{f} \end{array}$$

$$\begin{array}{c} \sum_{j=1}^{N}\lambda_{j}\phi (∥t_{0}-t_{j}∥)=\varphi_{1}\left(t_{0}\right), \end{array}$$

$$\begin{array}{c} \sum_{j=1}^{N}\gamma_{j}\phi (∥t_{0}-t_{j}∥)=\varphi_{2}\left(t_{0}\right). \end{array}$$

The purpose is to find $\Lambda =(\lambda_{1},\lambda_{2},\cdots ,\lambda_{N})$ and $\Gamma =(\gamma_{1},\gamma_{2},\cdots ,\gamma_{N})$ from Equation (18) such that minimize the cost function expressed in Equation (17). The described solution is called the RBF collocation method, developed as a set of MATLAB functions to transcribe the FOCP modeled in Equations (1) and (2) into an NLP optimization problem, and then use SNOPT [37] (i.e., a sparse NLP solver) to find the optimal trajectory. SNOPT uses a gradient-based optimization algorithm to solve the NLP, meaning that derivatives of cost and constraints must be provided. The proposed method has been developed in such a way that it automatically computes those gradients using the Symbolic Math Toolbox in MATLAB.

## 4. Numerical Implementation

Here, we apply the Cubic RBFs which is discussed in Section 3 for solving several DFOCPs. We test the performance of the proposed scheme on some test problems, and also present the results for different values of fractional order α and number of Cubic RBFs *N*. All numerical computations have been coded in Matlab R2015b on a 2.30 MHz Alpha Machine with 2GB RAM. Note that, in a minimization problem, the minimum value of the objective function is the best comparison to decide which the most efficient method is. This comparison between the proposed method and other previous algorithms can be found in the conclusion section. Moreover, comparison of these methods in terms of computational time (i.e., CPU time in seconds) is also provided along this section.

**Example** **1.** *Let us consider the first DFOCP as follows,*

(19) $$\min\;J=\frac{1}{2}\int_{0}^{2}\left(x^{2}\left(t\right)+u^{2}\left(t\right)\right)dt,$$

*subjected to the dynamical system*

$$\begin{array}{c} D^{\alpha }x\left(t\right)=x\left(t-1\right)+u\left(t\right),\;\;0<\alpha \leq 1,\\ x(t)=1,\;-1\leq t\leq 0, \end{array}$$

*where 0≤t≤2 and x(t)=0 at t<−1.*

**Figure 1:**
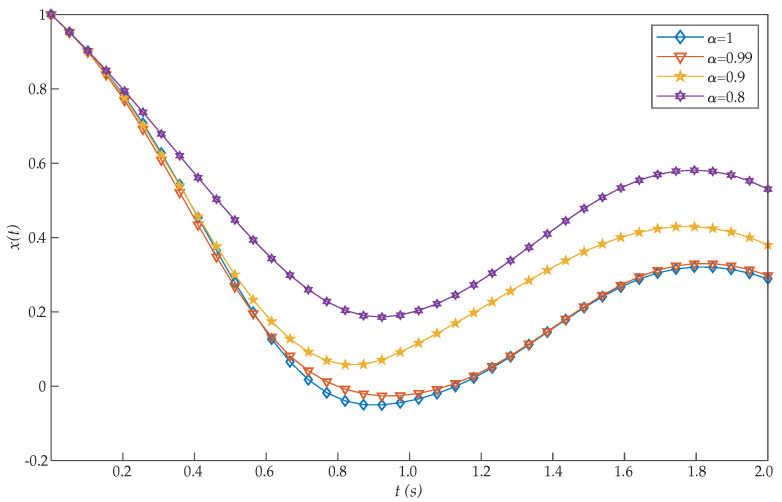
Evaluated function x(t) for the problem expressed in Equation (19).

**Figure 2:**
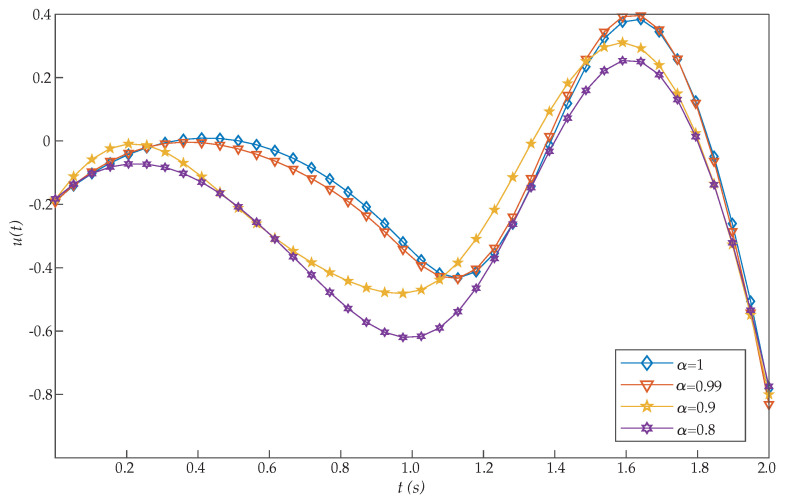
Evaluated function u(t) for the problem expressed in Equation (19).

This problem was introduced by Moradi and Mohammadi [38], who proposed a solution based on discrete Chebyshev polynomials. More precisely, the authors solved this problem for different choices of α [26,28]. Moreover, for α=1, Tohidi et al. [39] solved the problem using Müntz–Legendre spectral collocation method, and Ghomanjani et al. [40] used the Bezier curves for approximating the trajectory and control functions. However, the proposed RBF collocation method was more efficient than these and other previous algorithms, as Table 1 shows. From the perspective of cost values for various basis functions, our suggested approach is more effective by increasing *N*. Figure 1 and Figure 2 show the graphs of x(t) and u(t), respectively, for N=20. Moreover, these figures show that as α approaches 1, the solution for the integer order system is recovered.

In direct methods, initial guesses must be offered only for some quantities, like the states and possibly controls which are physically intuitive. As can be seen in Figure 1, the initial condition x(0)=1 is achieved with the proposed method. By contrast, that condition was not reached in previous works [26,28,38,39], thus increasing their error.

**Example** **2.** *Here, we consider the following FOCP with delay in control,*

(20) $$\min\;J=\frac{1}{2}\int_{0}^{\frac{1}{4}}\left(x^{2}\left(t\right)+u^{2}\left(t\right)\right)dt,$$

*subjected to the dynamical system*

$$\begin{array}{c} D^{\alpha }x\left(t\right)=x\left(t\right)+u\left(t-0.1\right)+u\left(t\right),\;\;0<\alpha \leq 1,\\ x(0)=1,\\ u(t)=0,\;-0.1\leq t\leq 0, \end{array}$$

**Figure 3:**
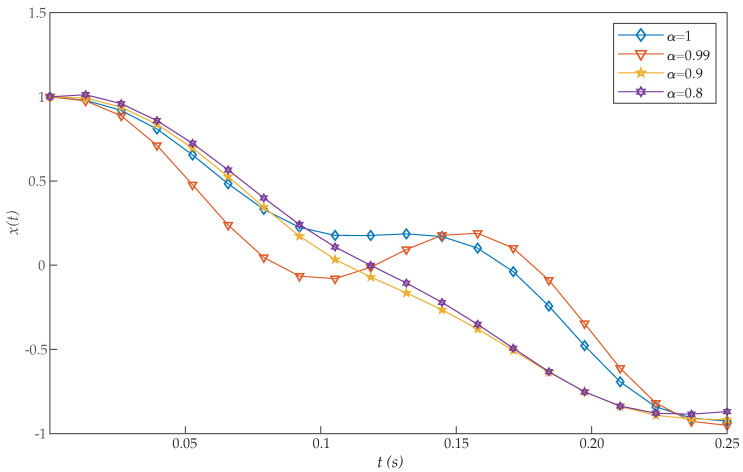
Evaluated function x(t) for the problem expressed in Equation (20).

**Figure 4:**
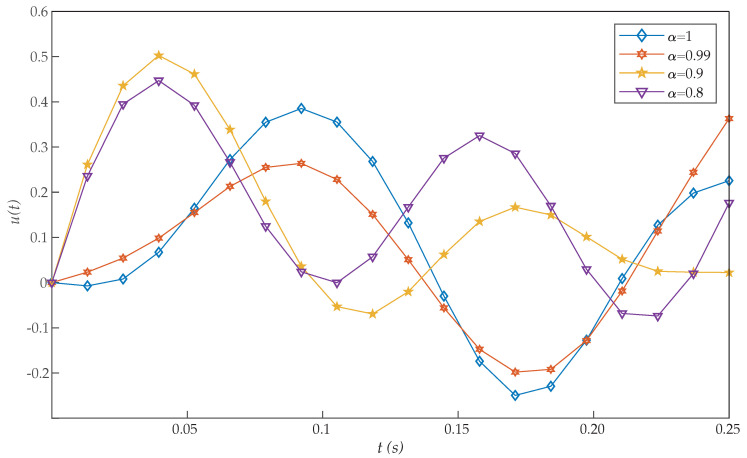
Evaluated function u(t) for the problem expressed in Equation (20).

*where $t\in [0,\frac{1}{4}]$. The values of J obtained by the proposed algorithm and other previous works [27,39,40] are presented in Table 2. As can be seen, the best performance was obtained by our approach, which also achieved good approximation results with small values of N. Figure 3 and Figure 4 displays the graphs of x(t) and u(t), respectively, for N=20. These figures corroborate the validity and efficacy of our method for this problem. Again, it can be seen that the initial condition x(0)=1 is achieved with the proposed method, while that condition was not obtained in other previous reports.*

**Example** **3.** *Consider a DFOCPs with two different delays in the form*

(21) $$\min\;J=\frac{1}{2}\int_{0}^{1}\left(x^{2}\left(t\right)+\frac{1}{2}u^{2}\left(t\right)\right)dt$$

*such that*

$$\begin{array}{c} D^{\alpha }x\left(t\right)=-x\left(t\right)+x\left(t-\frac{1}{3}\right)+u\left(t\right)-\frac{1}{2}u\left(t-\frac{2}{3}\right),\;0<\alpha \leq 1,\\ x\left(t\right)=1,\;-\frac{1}{3}\leq t\leq 0,\\ u\left(t\right)=0,\;-\frac{2}{3}\leq t\leq 0, \end{array}$$

**Figure 5:**
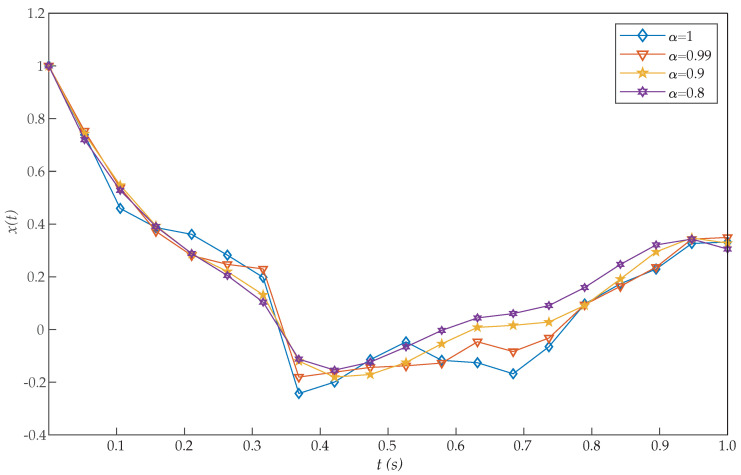
Evaluated function x(t) for the problem expressed in Equation (21).

**Figure 6:**
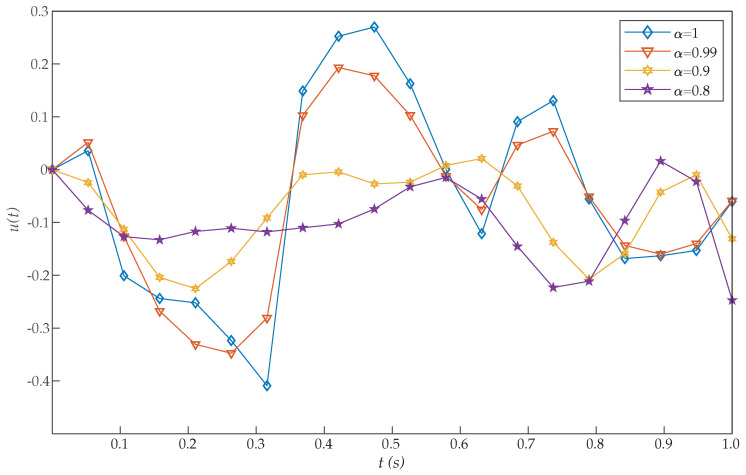
Evaluated function u(t) for the problem expressed in Equation (21).

*where 0≤t≤1. Table 3 shows the obtained values of J for α=1 with our scheme, Chelyshkov wavelets [27], Bernoulli polynomials [41], fractional-order Lagrange polynomials [42], Bernoulli wavelets basis [28], Müntz-Legendre polynomials [39], the least square method [40], and fractional-order Boubaker functions [29]. Again, the proposed algorithm also reported a very efficient performance. In addition, Table 4 illustrates the effect of the parameters α and N on the performance of the proposed method for this problem. In this case, we can see that good approximation results are also achieved by the proposed method with small values of N. The graphs of x(t) and u(t) with different values of α are shown in Figure 5 and Figure 6. It should be noted that, as α approaches 1, the numerical results converge to that of an integer-order differential equation. Moreover, the initial conditions x(0)=1 and u(0)=0 are achieved with the proposed method, while they were not reached in [28,29].*

**Example** **4.** *Consider the following time-varying DFOCP,*

(22) $$\min\;J=\int_{0}^{2}\left(x^{2}\left(t\right)+u^{2}\left(t\right)\right)dt,$$

*subject to:*

$$\begin{array}{c} D^{\alpha }x\left(t\right)=tx\left(t\right)+x\left(t-1\right)+u\left(t\right),\;0<\alpha \leq 1,\\ x(t)=1,\;-1\leq t\leq 0, \end{array}$$

**Figure 7:**
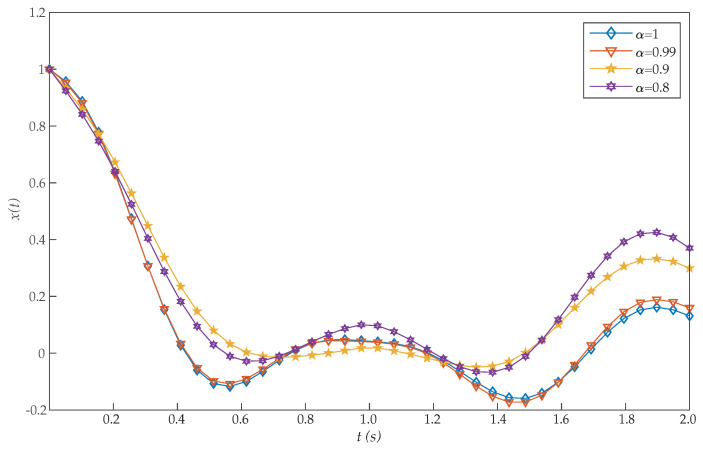
Evaluated function x(t) for the problem expressed in Equation (22).

**Figure 8:**
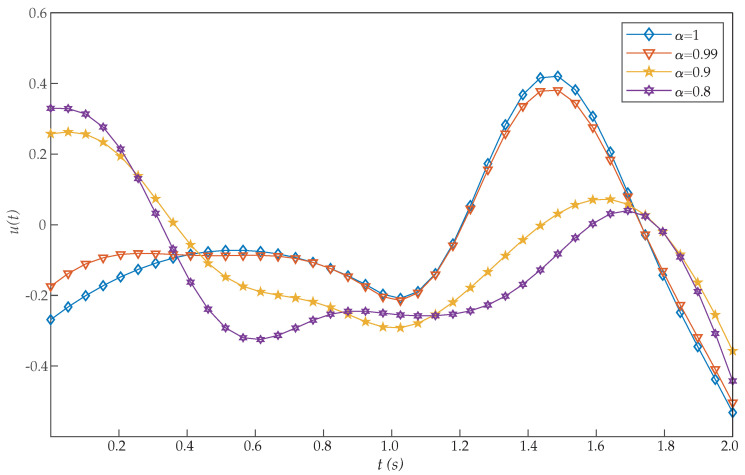
Evaluated function u(t) for problem expressed in Equation (22).

*where 0≤t≤2. This example have been solved by Rahimkhani et al. [28], Haddadi et al. [41], Ordukhani et al. [42], Moradi et al. [27,38], and Rabiei et al. [29], but any of them reached the initial condition x(0)=1. A comparison of the values of J obtained by these methods and that reported by the proposed scheme is presented in Table 5. Moreover, the effect of the parameters α and N on the proposed algorithm performance is displayed in Table 6. Both comparisons reveal that the accuracy of our method was higher than all previously proposed ones. Figure 7 and Figure 8 show the approximation graphs of x(t) and u(t) for N=20, respectively. We can see that, as α approaches 1, the numerical results converge to those obtained for an integer-order differential equation.*

**Example** **5.** *Consider the following DFOCP,*

(23) $$\min\;J=\int_{0}^{1}\left[X^{T}\left(t\right)\left(\begin{array}{cc} 1 & t\\ t & t^{2} \end{array}\right)X\left(t\right)+\left(t^{2}+1\right)u^{2}\left(t\right)\right]dt$$

*subject to:*

$$D^{\alpha }X\left(t\right)=\left(\begin{array}{cc} t^{2}+1 & 1\\ 0 & 2 \end{array}\right)X\left(t-\frac{1}{2}\right)+\left(\begin{array}{c} 1\\ t+1 \end{array}\right)u\left(t\right)+\left(\begin{array}{c} t+1\\ t^{2}+1 \end{array}\right)u\left(t-\frac{1}{4}\right),\;\;t\in \left[0,1\right]$$

*where $X\left(t\right)={\left[x_{1}\left(t\right)\;\;x_{2}\left(t\right)\right]}^{T}={\left[1,1\right]}^{T}$ for $-\frac{1}{2}\leq t\leq 0$ and u(t)=1, $-\frac{1}{4}\leq t\leq 0$. The exact solution of this problem is unavailable. Table 7 displays the numerical results achieved by the proposed method for various values of N and α=1, as well as for other previous algorithms dealing with the same problem. As can be seen, the obtained results corroborate the validity and efficacy of our method for this problem.*

## 5. Conclusions

This paper has introduced a new technique based on the collocation method to solve DFOCPs. The proposed design first uses collocation approximations of RBFs for control and state variables in the problem. In the next step, both the context of these basis functions and a joint application of the direct method allow us to turn a DFOCP into an NLP for finally choosing the coefficients and optimal control parameters. The numerical results obtained from several non-trivial examples, with a small number of *N* and some values of α, confirm the efficiency, accuracy, and high performance of the proposed approximation, which would remove ill-conditioning in most systems of discrete equations. Moreover, our results have also shown that using RBFs via a collocation method bears some advantages, such as simple evaluation of fractional derivatives and delay terms of given differential equations, and less expensive of computational costs. Moreover, as the necessary conditions need not be derived, the proposed direct method does not contain the difficulties of indirect approaches for DFOCPs. Consequently, other significant merits of the proposed approach are swift calculations, ease of implementation, and robustness. Indeed, it has provided satisfactory results when a small number of RBFs has been used. To this respect, comparison of cost values for different number of nodes discloses that the accuracy of the proposed RBF collocation method is higher than most previous methods, additionally requiring less CPU time (Please see Table 8).

## Figures and Tables

**Table 1:** Values of *J* and CPU time obtained by several algorithms for the problem expressed in Equation (19).

	Bhrawy	Moradi	Rahimkhani	Ghomanjani	Tohidi		This Study	
	[26]	[38]	[28]	[40]	[39]	N=5	N=15	N=20
*J*	0.472746	1.647883	0.3048	1.593587	1.647453	0.206476	0.110829	0.1101739
CPU Time (s)	–	3.265	–	–	4.358	–	–	2.02481

**Table 2:** Values of *J* and CPU time obtained by several algorithms for the problem modeled in Equation (20).

	Ghomanjani	Tohidi	Moradi		This Study	
α	[40]	[39]	[27]	N=5	N=15	N=20
1	0.156586	0.154268	0.1537475	0.176496	0.170357	0.177013
0.99	–	–	0.1539804	0.170073	0.165125	0.168257
0.9	–	0.159209	0.1560829	0.265759	0.244247	0.241156
0.8	–	0.163072	0.1584112	0.263194	0.251152	0.255855
CPU Time (s)	–	0.486	3.187	–	–	0.11579

**Table 3:** Values of *J* and CPU time obtained by several algorithms for the problem modeled in Equation (21).

	This Study	Haddadi [41]	Moradi [27]	Ordokhani [42]	Rahimkhani [28]	Tohidi [39]	Ghomanjani [40]	Rabiei [29]
*J*	0.061807	0.373112	0.373112	0.31851	0.1027	0.367700	0.422049	0.04553
CPU Time (s)	0.09601	–	3.125	0.141	–	25.559	–	–

**Table 4:** Values of *J* obtained by the proposed algorithm for the problem modeled in Equation (21) when different parameters α and N are analyzed.

*N*	α=1	α=0.99	α=0.9	α=0.8
5	0.068642	0.068672	0.067728	0.067632
10	0.062317	0.061347	0.060182	0.058799
15	0.062249	0.060186	0.056110	0.053929
20	0.061807	0.062287	0.057755	0.056017

**Table 5:** Values of *J* and CPU time obtained by several algorithms for the problem modeled in Equation (22).

	Haddadi	Rahimkhani	Rabiei	Ordokhani	Moradi		This Study	
	**[41]**	**[28]**	**[29]**	**[42]**	**[27]**	N=5	N=15	N=20
*J*	4.7407	2.0481	0.07762	2.0356	4.79679	0.194278	0.098498	0.096005
CPU Time (s)	–	–	–	0.094	3.640	–	–	0.06737

**Table 6:** Values of *J* obtained by the proposed algorithm for the problem modeled in Equation (22) when different parameters α and N are analyzed.

*N*	α=0.99	α=0.9	α=0.8
5	0.196364	0.220733	0.258642
10	0.122282	0.145960	0.170634
15	0.101511	0.128531	0.156858
20	0.096579	0.126915	0.152311

**Table 7:** Values of J obtained by several algorithms for the problem modeled in Equation (23).

	Rahimkhani	Ghomanjani	Wang		This Study	
	**[28]**	**[40]**	**[43]**	N=5	N=15	N=20
*J*	1.503157	1.536409753	1.562240664	1.509701	1.503127	1.501652
CPU Time (s)	–	–	–	–	–	8.156

**Table 8:** Summary of the values of *J* obtained by several algorithms for the tested problems with α=1.

Approximate Method	Example 1	Example 2	Example 3	Example 4	Example 5
Banks and Burns (1978) [44]	1.6419	−−	−−	−−	−−
Palanisamy and Rao (1983) [45]	1.6497	−−	−−	6.0079	−−
Dadebo and Luus (1992) [46]	−−	−−	−−	6.26775	−−
Chen et al. (2000) [47]	−−	−−	−−	4.7976	−−
Marzban and Razzaghi (2004) [48]	−−	−−	0.37311241	−−	−−
Basin and Gonzalez (2006) [49]	−−	0.1563	−−	−−	−−
Wang (2007) [43]	0.8512428	−−	0.37312	−−	1.562240664
Khellat (2009) [50]	−−	−−	−−	5.1713	−−
Haddadi et al. (2012) [41]	−−	−−	0.37310517	4.7407	−−
Ghomanjani et al. (2014) [40]	1.593587	0.15658669	0.4220497	−−	1.536409753
Safaie et al. (2014) [25]	0.6381	−−	0.3956	−−	−−
Safaie et al. (2014) [51]	1.0447	−−	−−	−−	−−
Bhrawy and Ezz-Eldien (2016) [26]	0.4727464	0.0143671	0.01451	−−	−−
Rahimkhani et al. (2016) [28]	0.3048	−−	0.1027	2.0481	1.503157
Jajarmi et al. (2017) [52,53]	1.64886527	−−	−−	4.79678	−−
Rabiei et al. (2017) [29]	0.00002674	−−	0.04553	0.07762	−−
Moradi et al. (2018) [27]	1.64787419	0.15374756	0.37311264	4.79679868	−−
Tohidi et al. (2019) [39]	1.647453	0.154268	0.367700	−−	−−
Present method	0.1101739	0.177013	0.061807	0.096005	1.501652
